# Measles in conflict-affected northern Syria: results from an ongoing outbreak surveillance program

**DOI:** 10.1186/s13031-021-00430-0

**Published:** 2021-12-21

**Authors:** Sammy Mehtar, Naser AlMhawish, Kasim Shobak, Art Reingold, Debarati Guha-Sapir, Rohini J. Haar

**Affiliations:** 1 UC Berkeley – UCSF Joint Medical Program, Berkeley, USA; 2 Assistance Coordination Unit, Gaziantep, Turkey; 3 Division of Epidemiology and Biostatistics, School of Public Health, University of California, Berkeley, Berkeley, CA 94705 USA; 4 Centre for Research on the Epidemiology of Disasters, University of Louvain School of Public Health, Leuven, Belgium

**Keywords:** Syria, Conflict, War, Measles, Epidemic, Infectious diseases, Surveillance, Vaccine, Vaccine-preventable disease

## Abstract

**Background:**

The Syrian conflict has dramatically changed the public health landscape of Syria since its onset in March of 2011. Depleted resources, fractured health systems, and increased security risks have disrupted many routine services, including vaccinations, across several regions in Syria. Improving our understanding of infectious disease transmission in conflict-affected communities is imperative, particularly in the Syrian conflict. We utilize surveillance data from the Early Warning Alert and Response Network (EWARN) database managed by the Assistance Coordination Unit (ACU) to explore trends in the incidence of measles in conflict-affected northern Syria and analyze two consecutive epidemics in 2017 and 2018.

**Methods:**

We conducted a retrospective time-series analysis of the incidence of clinically suspected cases of measles using EWARN data between January 2015 and June 2019. We compared regional and temporal trends to assess differences between geographic areas and across time.

**Results:**

Between January 2015 and June 2019, there were 30,241 clinically suspected cases of measles reported, compared to 3193 cases reported across the whole country in the decade leading up to the conflict. There were 960 regional events that met the measles outbreak threshold and significant differences in the medians of measles incidence across all years (p-value < 0.001) and in each pairwise comparison of years as well as across all geographic regions (p-value < 0.001). Although most governorates faced an elevated burden of cases in every year of the study, the measles epidemics of 2017 and 2018 in the governorates of *Ar-Raqqa*, *Deir-Ez-Zor, and Idlib* accounted for over 71% of the total suspected cases over the entire study period.

**Conclusions:**

The 2017 and 2018 measles epidemics were the largest since Syria eliminated the disease in 1999. The regions most affected by these outbreaks were areas of intense conflict and displacement between 2014 and 2018, including districts in *Ar-Raqqa*, *Deir-Ez-Zor*, and *Idlib*. The spread of measles in northern Syria serves as an indicator of low immunization coverage and limited access to care and highlights the Syrian peoples’ vulnerability to infectious diseases and vaccine preventable diseases in the setting of the current conflict.

**Supplementary Information:**

The online version contains supplementary material available at 10.1186/s13031-021-00430-0.

## Introduction

Armed conflict is a major cause of death and disability worldwide [1–5]. Some of the effects of armed conflict on health are direct, such as traumatic injuries and deaths among civilians and combatants, while others are indirect, such as disrupted health systems, displaced populations, a reduced health workforce, the breakdown of infrastructure, and heightened risk of disease transmission. The impact of indirect consequences is more difficult to quantify but disproportionately contributes to morbidity and mortality [1, 2, 4, 6].

The Syrian conflict began in March of 2011 as peaceful protests against the Syrian government in the context of the Arab Spring. These protests were violently suppressed, and clashes continued to escalate into armed conflict over the next year. Many different opposition groups emerged throughout the conflict, and a complex web of shifting conflict and alliances emerged. Between 2011 and 2015 the Syrian government lost control of much of north and north-east Syria, but with increased foreign support the Syrian government gradually regained control of most of these territories over the next five years.

Since the Syrian conflict’s onset in 2011, the Syrian people have suffered significant health challenges including targeted attacks on healthcare facilities, healthcare workers, and patients, lack of medical supplies, disease outbreaks, and the disruption of preventative services [4, 7–15]. These challenges, coupled with massive inflation, limited supplies, energy shortages, lack of safe transportation, loss of vital infrastructure (e.g., water, sanitation, hospitals), and the displacement of over half of Syria’s physicians have led to dramatic changes in the public health landscape of conflict-affected Syria [16–21]. There has also been dramatic population shifts: out of an estimated pre-war population of 22 million, approximately 12.2 million people (55.5% of the pre-war population) have been displaced as either a refugee (5.6 million people, 45.9% of the displaced population) or as an internally displaced person (6.6 million, 54.1% of the displaced population) [22–24]. These mass internal migrations, typically to urban centers such as Idlib, coupled with damaged infrastructure and housing, have resulted in overcrowding that renders these regions vulnerable to communicable diseases.

Syria’s public health system has taken a particular toll in regions that fell outside of government control where the Syrian Ministry of Health could no longer operate or monitor health [25, 26]. Reports indicate that the main contributors to morbidity in children under five years have been infectious diseases and malnutrition [27]. However, little is known about how the war has shaped the epidemiology of vaccine-preventable diseases, especially in territories beyond the reach of the Syrian Ministry of Health.

Measles is particularly important in the Syrian context. It has a basic reproductive number (R_0_) of 12–18, making it one of the most infectious diseases known. As a comparison, SARS-CoV-2 has an R_0_ of 1.8–3.6 [28]. Measles is acutely sensitive to drops in vaccination coverage; the effects of changes in vaccination coverage on measles incidence can be seen within one to two years, making it an early indicator of declining vaccination rates [29–32]. Children under five years of age are at the highest risk for symptomatic infection and complications [30]. The measles vaccine is generally administered in a two-dose schedule and is 97% effective when both doses are given; however, ensuring that those who are susceptible receive both doses adds to the logistical challenge of vaccination [30, 33]. Measles can also lead to severe complications, which, although relatively rare, disproportionately affect children under five years of age. Measles case fatality ratios range from 0.1 to 5%, depending on the age of infection, nutrition status, vaccine coverage, and access to healthcare, factors all relevant to the Syrian context [18, 20, 30]. Communities that have higher than 90% vaccination rates are considered to have “herd immunity” and are less likely to experience outbreaks [30, 32].

Pre-conflict data from 1999 onwards illustrates that Syria had maintained relatively high vaccination coverage and low measles incidence, although surveillance was unreliable and there is limited access to this data [34–36]. Since the conflict started, health officials have attributed the spread of preventable diseases, including measles, to diminished preventative services as well as uncoordinated and delayed response efforts in those areas [9, 15, 37]. Even before the conflict, by at least 2010, “the government stopped maintaining sanitation and safe-water services, and began withholding routine immunizations for preventable childhood diseases” in regions “considered politically unsympathetic [38],” rendering those regions even more susceptible to preventable diseases. A Type-1 wild poliovirus (WPV-1) outbreak in 2013 highlighted significant weaknesses in the health system [26, 39, 40].

This study aims to describe the incidence of measles in conflict-affected Syria and explore associations between measles incidence and conflict indicators. We use an infectious disease surveillance dataset to study the epidemiology of measles, analyze its relationship to conflict and displacement, and illustrate geographic and chronological trends [41]. We hope this data will contribute to broader understanding of infectious disease vulnerabilities in conflict and lead to more attention, resources and support for the regions most affected, while highlighting the importance of vaccines as a human right.

## Methods

We conducted a secondary analysis of infectious disease surveillance data about conflict-affected northern Syria between January 1st, 2015, and July 31st, 2019, collected by the Early Warning Alert and Response Network (EWARN) operated by the Assistance Coordination Unit (ACU). We describe the EWARN surveillance protocol, the relevant geopolitical context, and then review the study analysis.

### EWARN surveillance system

In September 2012, the World Health Organization (WHO), in collaboration with the Syrian Ministry of Health, established the Early Warning and Response System (EWARS) to improve surveillance efforts [42]. EWARS was designed for rapid and cost-effective implementation in humanitarian or conflict settings to improve detection of infectious disease outbreaks [40, 42, 43]. However, it was limited to regions under government control. The 2013 polio outbreak in northern Syria motivated the development of an additional surveillance effort, the Assistance Coordination Unit’s (ACU) system called the Early Warning Alert and Response Network (EWARN) [39]. EWARN is an active surveillance program that operates in regions outside of Syrian government control. EWARN collects data on 13 diseases and conditions, selected for their potential to cause epidemics, their association with high morbidity and mortality, and the potential for intervention in Syria [40]. There is no overlap between EWARS and EWARN coverage areas.

As a syndromic infectious disease surveillance system, EWARN reports clinically suspected cases. Suspected cases are not all laboratory-confirmed; instead, they meet clinical case definitions [40, 44]. Clinically suspected cases is the measure most heavily relied upon by response organizations and what is reported by the WHO when widespread laboratory testing is not available. In the case of measles, between 2015 and 2019, the ACU used the WHO definition: suspected cases are those in which the patient presents with a fever and non-vesicular maculopapular rash, or in whom a healthcare worker suspects measles [34, 40, 44]. Clinically compatible cases are those in which patients present with fever and a maculopapular rash and at least one of the following symptoms: cough, coryza or conjunctivitis [34]. Data are reported weekly.

Outbreaks are defined by the ACU using a modified WHO definition: greater than or equal to five clinically suspected cases that are temporally related in a district. The WHO definition is “two or more laboratory-confirmed cases that are temporally related (with dates of rash onset occurring 7–23 days apart) and epidemiologically- or virologically-linked, or both [45].”

EWARN tests the specificity of its syndromic surveillance. The ACU investigates districts with measles outbreaks, and part of the investigation process includes randomly selecting 5–10 clinically suspected cases of measles in each affected community for laboratory confirmation. Venous blood samples taken from clinically suspected patients at first contact with the healthcare system are sent for IgM ELISA testing. Between 65 and 80% of samples from suspected cases sent to laboratories for testing were positive for measles, resulting in 20–35% of laboratory tested cases reclassified as “discarded”.

We received the data as aggregated weekly case counts stratified by age groups (≤ 5 years of age, > 5 years of age), sex (male, female), and subdistrict the suspected cases were reported in. No personally identifiable data was included, and subdistrict level of localization was chosen to avoid potential for case-tracing and protect the identity of patients.

### Study design and sampling

*Geographical dimensions:* The Syrian conflict has developed into a complex, international conflict with many actors, factions, and proxies, often with competing interests. The political landscape in Syria has been dynamic throughout the conflict. As the geopolitical realities have shifted, so, too, have the coverage regions of EWARN and EWARS. For this analysis, the complex geopolitical landscape was simplified into two categories: territories that were under the control of the Syrian government, referred to as government-held territories, and those not in the control of the Syrian government, referred to as opposition-controlled territories. We focused exclusively on EWARN data from opposition-controlled territories. EWARS is similar in methods and scope but is managed directly by WHO and operates in regions controlled by the Government of Syria.

Syria is administratively divided into 14 governorates (*muhafazat*), which are further divided into 65 districts (*manatiq*) and 281 subdistricts (*nawahi*). The governorates of Damascus, Rural Damascus, and Lattakia, and the districts of *As-Safira*, *Tadmor*, and *Al-Fiq* were excluded from this analysis because they have remained outside of EWARN’s coverage region for most, if not all, of the conflict. The other 11 governorates and constituent districts were included because they remained within the coverage region, with a few exceptions. Districts that start as opposition-controlled territories that then fall out of coverage after becoming government-held territories are reported as having missing case reports. We only included districts that remained within the coverage region for at least 4 of the 4.5 years of the study. While the ACU collects data at the community level, limitations in population estimates for 2015–2016 and ongoing security concerns compelled us to restrict our analysis to the district level.

**Fig. 1:**
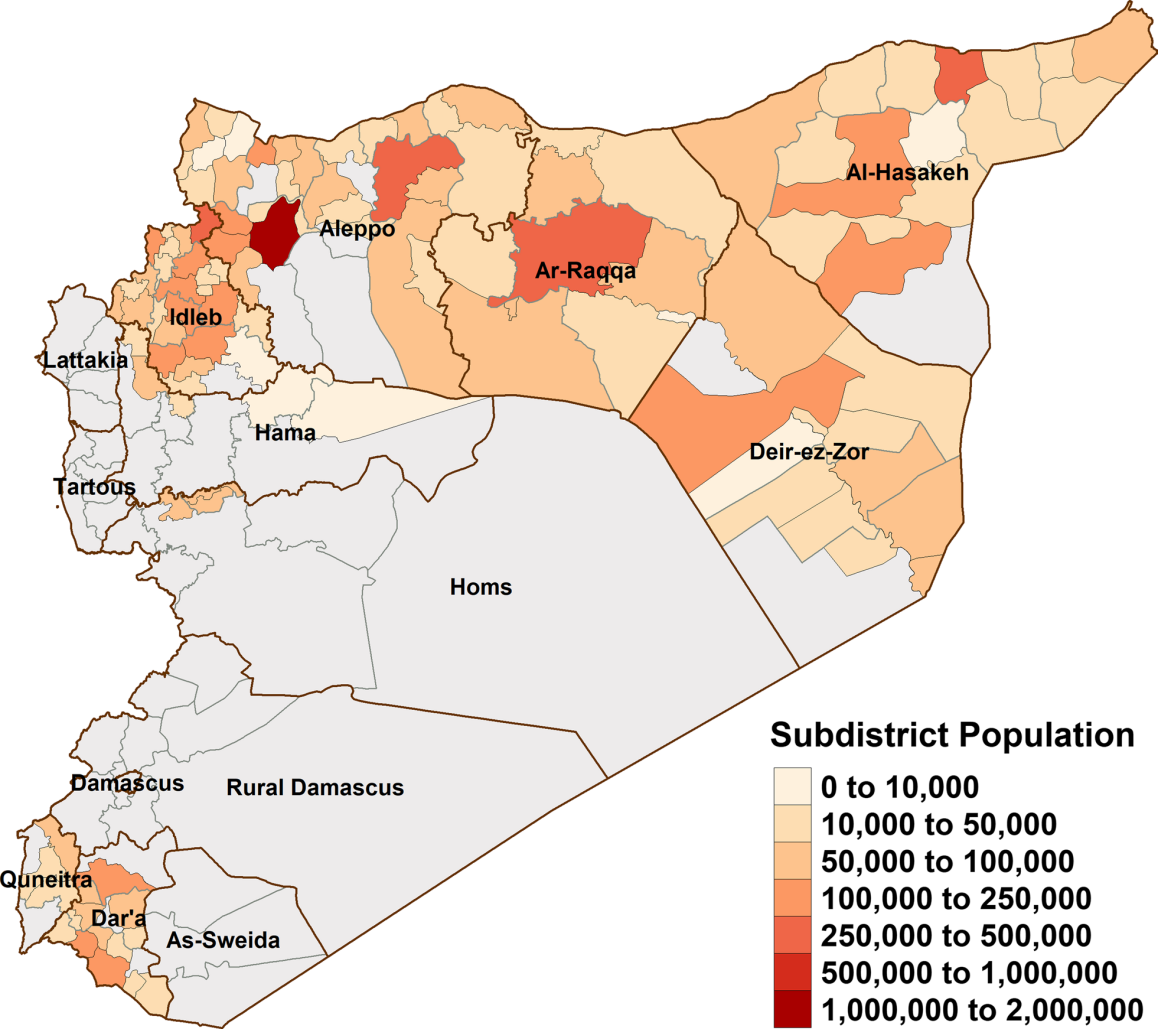
Population by subdistrict, 2017

Population estimates for Syria between 2015 and 2019 were obtained from the Humanitarian Needs Assessment Programme (HNAP) of the United Nations Office for the Coordination of Humanitarian Affairs (UNOCHA) [46, 47]. These population estimates are made monthly by collecting data using questionnaires and interviews, consulting key informants, and making observations about population movement. Population estimates are then distributed to United Nations-affiliated agencies and other governmental and non-governmental organizations (NGOs) working in Syria. From 2015 to 2016, population estimates made at the district level, while from 2017 to 2019 population estimates were made at the subdistrict-level. Thus, we limited our estimates of incidences to the district-level, despite subdistrict-level granularity of the surveillance data for a portion. Figure 1 illustrates subdistrict population estimates from 2017 (Additional file 1).

### Statistical analysis

We conducted descriptive analyses of surveillance data to report on the study population characteristics, which included binary variables for age, sex, and categorical variables for geographic district.

The data was found to not be normally distributed using the Shapiro Wilk normality test (P < 0.001) and therefore classified as non-parametric. The Wilcoxon rank-sum test and Kruskal–Wallis test were used for significance testing, using the Benjamini–Hochberg procedure for p-value adjustment, with a P-value of < 0.05 chosen as the threshold for significance to test whether differences in the distribution of weekly incidence between different districts were significant. Data were stored and shared using Microsoft Excel. The results were analyzed and visualized using R.

*Ethical approval.*

This study was reviewed and deemed exempt by the Committee for the Protection of Human Subjects at the University of California, Berkeley.

## Results

### Overall surveillance

There was a total of 40,577,249 visits to facilities within the EWARN during the period of January 1, 2015, to June 30, 2019. Of those, 7,925,079 (19.5%) visits met the criteria for one of the EWARN syndromes.

### Descriptive analysis of measles epidemiology

**Fig. 2:**
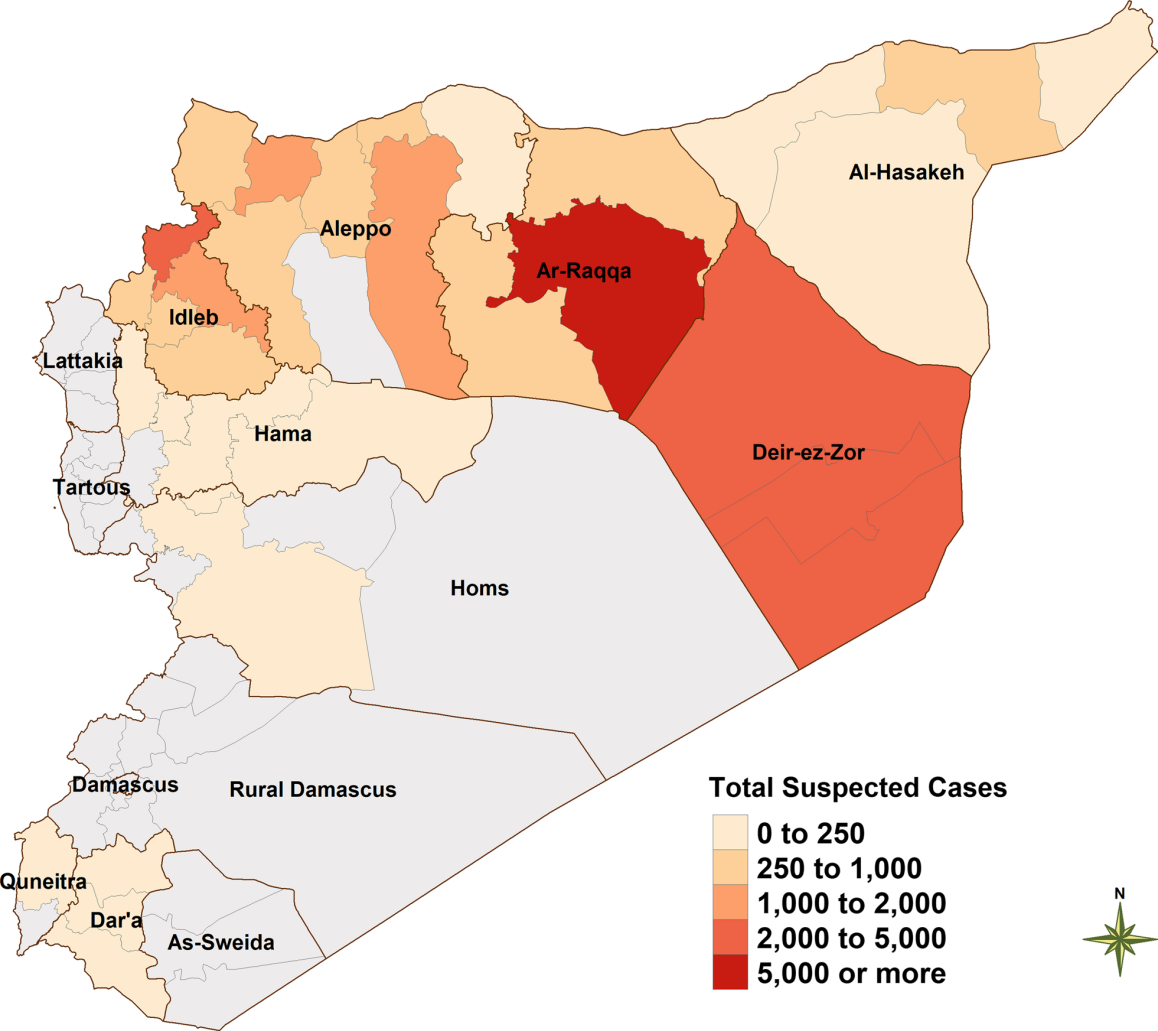
Total number of suspected cases of measles January 2015–2019

A total of 30,241 suspected cases of measles were reported in opposition-controlled territories during the 4.5-year study period (Table 1). Suspected cases of measles were reported in every governorate except for Homs. The governorates of *Deir-Ez-Zor* (n = 9027, 29.9% of suspected cases) and *Ar-Raqqa* (n = 8786, 29.1% of suspected cases) had the highest numbers of total suspected cases during the study period, followed by Aleppo (n = 5763, 19.1% of suspected cases) and *Idlib* (n = 5531, 18.3% of suspected cases) (Fig. 2). Depending on the availability of testing and security conditions, between 65 and 80% of clinically suspected cases of measles that were tested were confirmed as true cases using serologic testing [40].

**Table 1:** Suspected cases of measles by district, northern Syria, January 2015–June 2019

Districts	2015	2016	2017	2018	2019*	Total cases
*Aleppo total*	651	500	1912	2612	88	5763
Afrin	7	6	110	180	4	307
Ain Al Arab	18	14	11	33	0	76
Al Bab	67	30	107	484	7	695
A'zaz	16	132	1004	687	32	1871
Jarablus	18	28	126	337	15	524
Jebel Saman	83	22	9	774	28	916
Menbij	442	268	545	117	2	1374
*Al-Hasakeh total*	389	59	37	129	9	623
Al-Hasakeh	42	6	6	6	1	61
Al-Malikeyyeh	54	1	11	37	7	110
Quamishli	177	12	13	67	1	270
Ras Al Ain	116	40	7	19	0	182
*Ar-Raqqa total*	655	908	669	6512	42	8786
Ar-Raqqa	512	816	486	5741	30	7585
Ath-Thawrah	136	81	101	397	5	720
Tell Abiad	7	11	82	374	7	481
*Dar'a total*	20	27	164	56	*NA*	267
As-Sanamayn	2	0	4	2	*NA*	8
Dar'a	17	14	88	26	*NA*	145
Izra'	1	13	72	28	*NA*	114
*Deir-ez-Zor total*	280	414	4204	4070	59	9027
Abu Kamal	138	110	2100	153	0	2501
Al Mayadin	51	83	1532	607	0	2273
Deir-ez-Zor	91	221	572	3310	59	4253
*Hama total*	20	9	7	56	1	93
As-Salamiyeh	9	5	1	2	*NA*	17
As-Suqaylabiyah	11	4	6	24	1	46
Hama	0	0	0	24	*NA*	24
Muhradah	0	0	0	6	0	6
*Homs total*	0	1	0	0	*NA*	1
Ar-Rastan	0	1	0	0	*NA*	1
Homs	0	0	0	0	*NA*	0
*Idleb total*	175	188	633	4444	191	5631
Al Ma'ra	38	21	39	323	14	435
Ariha	21	13	57	158	6	255
Harim	47	52	227	2061	98	2485
Idleb	53	54	211	1530	69	1917
Jisr-Ash-Shugur	16	48	99	372	4	539
*Quneitra total*	2	4	38	6	*NA*	50
Quneitra	2	4	38	6	*NA*	50
Total	2192	2110	7664	17,885	390	30,241

Within the coverage region, children less than 5 years of age accounted for 60.9% (n = 18,416) of reported cases of measles. While there was no significant difference between males under the age of 5 years and females under the age of 5 years (females < 5 years: n = 15,544, 51.4% vs. males < 5 years: n = 14,697, 48.6%, respectively, Wilcoxon Rank Sum Test p-value = 0.726), there was a statistically significant difference between sexes in cases ≥ 5 years of age (females ≥ 5 years n = 16,481, 54.5% vs. males ≥ 5 years n = 13,729, 45.4% of ≥ 5 cases, respectively, Wilcoxon Rank Sum Test p-value < 0.001), with more cases among females than in males.

### Measles epidemics

**Fig. 3:**
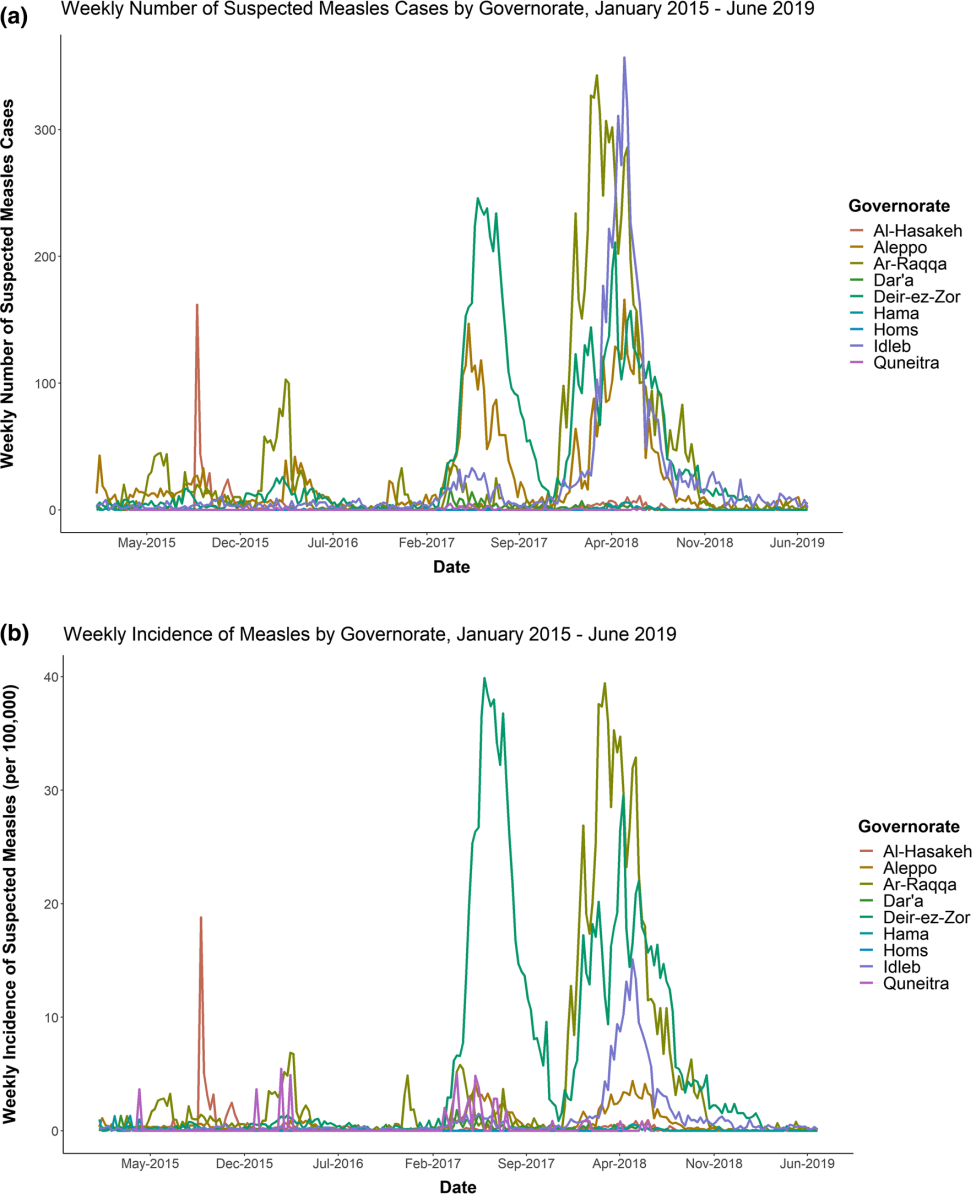
**a** Weekly number of suspected measles cases by governorate, January 2015–June 2019. **b** Weekly incidence of suspected measles cases, January 2015–June 2019

Using current measles outbreak threshold of ≥ 5 cases per district per month, 960 episodes met the outbreak threshold throughout the study period. However, several stood out visually as especially severe in terms of the absolute number of suspected cases, the incidence, and the longevity of the outbreaks (Fig. 3a and b).

The highest annual incidences of measles occurred in 2017 and 2018. In 2017, an epidemic localized to *Deir-Ez-Zor* governorate began in late March, with a rapid escalation to peak incidence levels in June and early July, and a gradual return to baseline incidence levels by late October. The 2018 epidemic, localized to the neighboring *Ar-Raqqa* governorate, began earlier, in November 2017, and lasted nearly a year, until the beginning of October 2018.

### Geographic distribution of epidemics/outbreaks

**Fig. 4:**
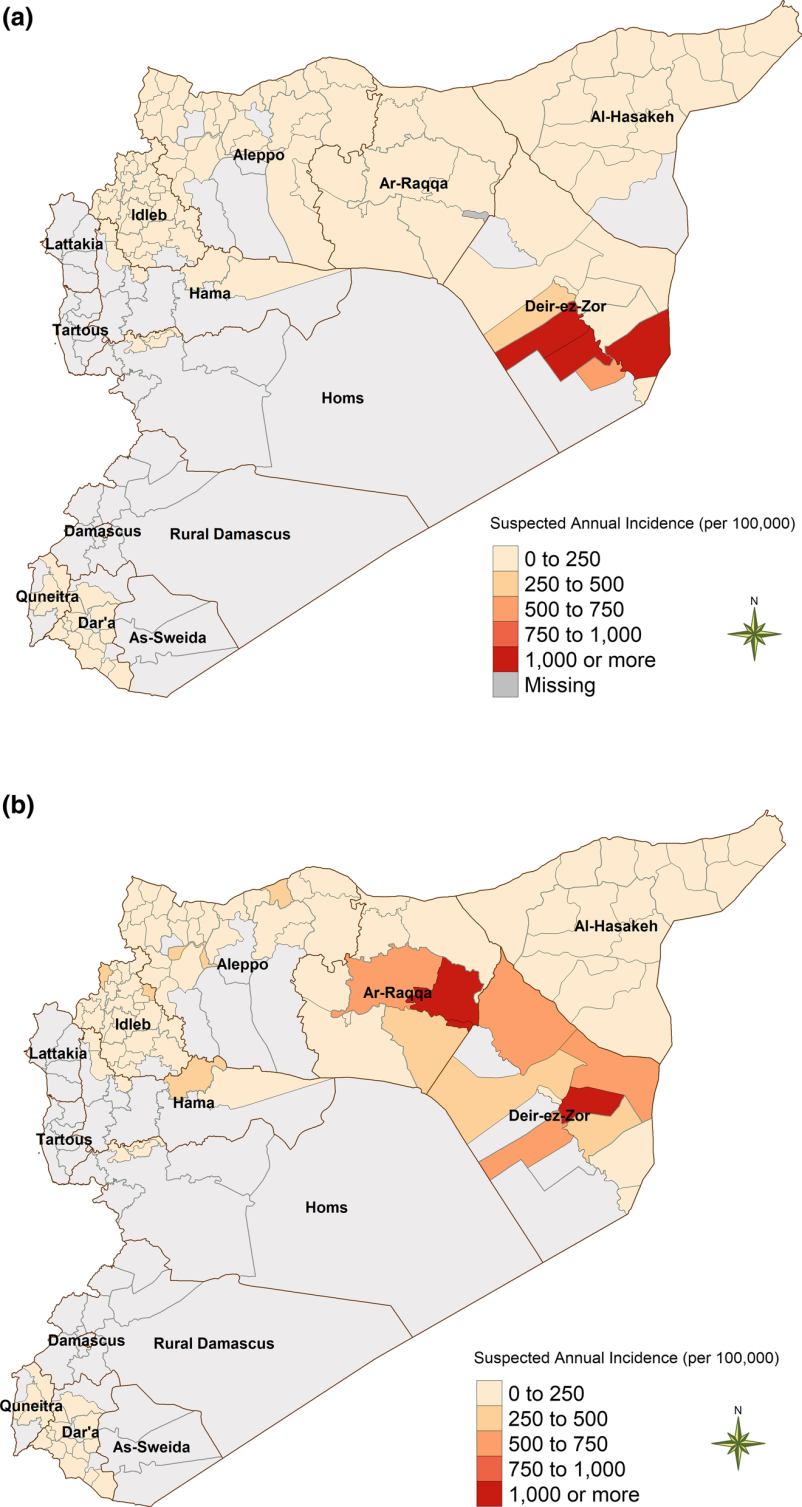
**a** Annual incidence of measles in Syria at the subdistrict level, 2017. **b** Annual incidence of measles in Syria at the subdistrict level, 2018

The measles epidemic in 2017 was primarily confined to *Abu Kamal* (1050 cases per 100,000 person-years) and *Al-Mayadin* (2138 cases per 100,000 person-years), two neighboring districts in the east of the country in the *Deir-Ez-Zor* governorate, as well as an outbreak in *A’zaz*, in the north of the Aleppo governorate (Fig. 4a). In 2018, the epidemic spread farther west and affected more populated regions, including the districts of *Al-Mayadin* (873 cases per 100,000 person-years) and *Deir-Ez-Zor* (691 cases per 100,000 person-years) in the *Deir-Ez-Zor* governorate as well as *Ar-Raqqa* (1471 cases per 100,000 person-years), *Ath-Thawrah* (359 cases per 100,000 person-years), and *Tell Abiad* (499 cases per 100,000 person-years) districts in the *Ar-Raqqa* governorate and the *Harim* (460 cases per 100,000 person-years) and *Idleb* (513 cases per 100,000 person-years) districts of *Idleb* governorate (Fig. 4b). There was also an outbreak in *Jarablus* (749 cases per 100,000 person-years) in the north of the Aleppo governorate. Districts with a high incidence of measles in 2017 experienced a decrease in incidence the following year, and no outbreaks were reported in 2019, suggesting a reduction in the at-risk population due to exhaustion of susceptible persons and vaccination efforts.

The two districts most affected by the 2017 epidemic, *Abu Kamal* and *Al-Mayadin*, experienced 2100 suspected cases and 1532 suspected cases, respectively, collectively accounting for 3632 of the 7644 suspected cases (47.5%) for 2017, despite their smaller populations. In 2018, the top four districts affected by the epidemic accounted for a total of 14,020 suspected cases of the 17,885 suspected cases that year (78.4%), including 5741 suspected cases in *Ar-Raqqa* district, 3310 suspected cases in *Deir-Ez-Zor* district, 2061 suspected cases in *Harim* district, and 1530 suspected cases in the *Idleb* district.

These two outbreaks were the most significant contributors to the 30,241 suspected cases of measles during the study period, accounting for 84.4% of all suspected cases. Relatively fewer suspected cases, 2192 (7.2%) and 2110 (7.0%), were documented in 2015 and 2016 respectively. In contrast, 2017 had a total of 7664 suspected cases (25.3%), while 2018 had 17,885 suspected cases (59.1%). The first half of 2019 had 390 suspected cases (1.29%).

Although districts within the governorates of *Ar-Raqqa* and *Deir-Ez-Zor* were most affected, a substantial number of cases occurred throughout the study period in other governorates as well, including Aleppo (n = 5763, 19.1% of all cases) and *Idlib* (n = 5631, 18.6% of all cases).

### Statistical analysis of overall incidence

There was a statistically significant difference between the incidence of suspected cases of measles in the < 5-year-old population when compared with the ≥ 5 year-old population over the entire study period, despite the smaller population size of the < 5-year-old population (Table 2). This difference is expected since children and infants are at the highest risk for measles.

**Table 2:** Wilcoxon Rank-Sum Test comparing incidence of suspected measles cases by bivariate age, and then by sex

Wilcoxon 2 × 2	p-value	W
< 5 vs. ≥ 5	< 2.2 × 10^−16^	2,526,055
Male vs. female	0.0581	23,364,969

**Fig. 5:**
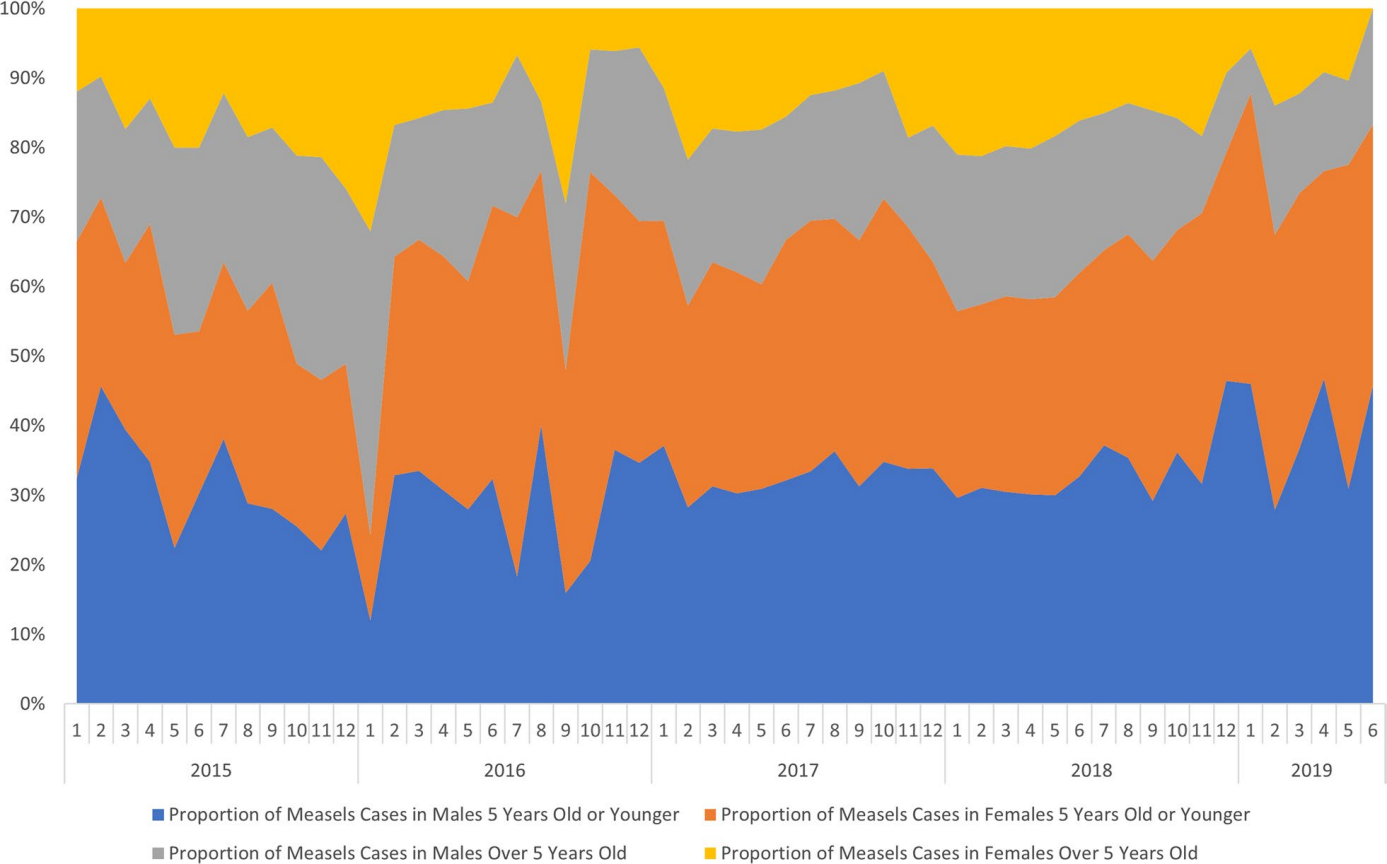
Relative proportion of measles cases by age and gender

There was no statistically significant difference in the incidence of suspected cases between males and females over the entire study period, but when stratifying by age, we found that there was a statistically significant difference between males and females ≥ 5 years of age, with higher incidence in males (Table 3). Figure 5 shows the relative proporion of cases by age and sex. Notably, there is a higher proportion of children under five years of age during the last two years of our dataset.

**Table 3:** Wilcoxon Rank-Sum Test comparing incidence of suspected measles cases stratified by age and sex

Wilcoxon 4 × 4	p-value	W
Male < 5 vs. female < 5	0.726	22,949,567
Male ≥ 5 vs. female ≥ 5	2.69 × 10^−5^	23,655,744
Male < 5 vs. male ≥ 5	< 2.2 × 10^−16^	24,479,876
Female < 5 vs. female ≥ 5	< 2.2 × 10^−16^	25,193,455

### Measles incidence over time

The incidence of suspected measles between the years was compared using the Kruskal–Wallis rank-sum test. The test had four degrees of freedom and yielded X^2^ = 569.39 with a p-value of < 0.001. Pairwise Wilcoxon rank-sum testing was done to compare each year to every other year. Only the years 2016 and 2019 (where we had incomplete data) were not significantly different from each other; all other pairings of years were significantly different (Table 4).

**Table 4:** Adjusted P-values of pairwise Wilcoxon Rank-Sum Tests comparing the distribution of weekly incidence of measles between each year

Year	2015	2016	2017	2018
2016	1.9 × 10^−5^	–	–	–
2017	6.4 × 10^−11^	< 2.2 × 10^−16^	–	–
2018	< 2.2 × 10^−16^	< 2.2 × 10^−16^	< 2.2 × 10^−16^	–
2019	1.9 × 10^−5^	0.27	< 2.2 × 10^−16^	< 2.2 × 10^−16^

### Measles incidence across geography

The Kruskal–Wallis rank-sum test was also used to compare the incidence of suspected measles between governorates. This test had eight degrees of freedom and yielded X^2^ = 1253.5 with a p-value < 0.001. Similar pairwise testing was done to compare incidence of each governorate to every other governorate. Differences in incidence rates between each governorate were all significant, except for *Al-Hasekeh*¸ *Dar’a*, and *Quneitra* which were found not to be significantly different (Table 5).

**Table 5:** Adjusted P-values of pairwise Wilcoxon Rank-Sum Tests comparing the distribution of weekly incidence of measles between each governorate over the study period

Governorate	*Al-Hasakeh*	*Aleppo*	*Ar-Raqqa*	*Dar’a*	*Deir-ez-Zor*	*Hama*	*Homs*	*Idleb*
*Aleppo*	< 2 × 10^−16^	–	–	–	–	–	–	–
*Ar-Raqqa*	< 2 × 10^−16^	3.8 × 10^−7^	–	–	–	–	–	–
*Dar’a*	0.0682	< 2 × 10^−16^	< 2 × 10^−16^	–	–	–	–	–
*Deir-ez-Zor*	< 2 × 10^−16^	< 2 × 10^−16^	4.0 × 10^−4^	< 2 × 10^−16^	–	–	–	–
*Hama*	1.3 × 10^−13^	< 2 × 10^−16^	< 2 × 10^−16^	< 2 × 10^−16^	< 2 × 10^−16^	–	–	–
*Homs*	< 2 × 10^−16^	< 2 × 10^−16^	< 2 × 10^−16^	< 2 × 10^−16^	< 2 × 10^−16^	4.6 × 10^−6^	–	–
*Idleb*	< 2 × 10^−16^	0.0180	0.0180	< 2 × 10^−16^	< 2 × 10^−16^	< 2 × 10^−16^	< 2 × 10^−16^	–
*Quneitra*	0.287	2.1 × 10^−11^	8.6 × 10^−16^	0.0374	< 2 × 10^−16^	7.4 × 10^−4^	3.9 × 10^−11^	< 2 × 10^−16^

## Discussion

This study focused on a descriptive and statistical analysis of the incidence of measles from January 1, 2015, through June 30, 2019, in conflict-affected northern Syria using data from an active surveillance system program in opposition-controlled territories. We found that measles, which had been eliminated in Syria in 1999, has seen a resurgence, with major epidemics in 2017 and 2018, and a total of 30,241 suspected cases identified in opposition-controlled territories in less than five years [48]. This statistic alone illustrates the scope of the breakdown of health system amidst the Syrian conflict.

The 30,241 suspected cases were distributed across 960 outbreaks. Large, extended outbreak events suggest that there were significant gaps in immunization such that herd immunity did not protect at least some districts in northern Syria, while the high number of outbreak events might suggest repeated reintroductions from nearby communities.

**Fig. 6:**
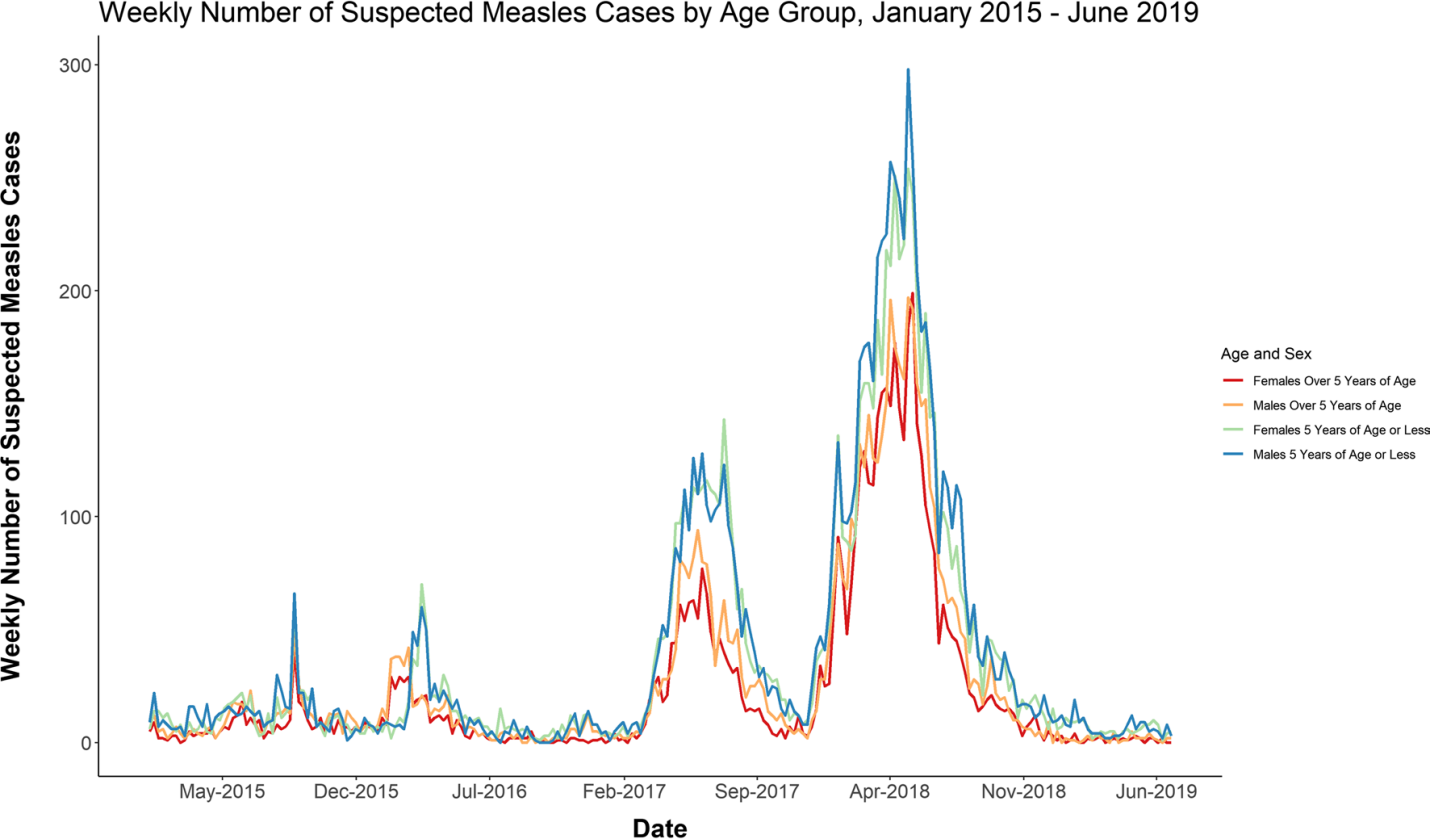
Weekly number of suspected measles cases by age group, January 2015–2019

Although the districts affected by the epidemic in 2017 had higher annual incidence rates reaching as high as 2000 cases per 100,000 person-years, the absolute number of cases was higher in 2018, because the districts affected by the epidemic in 2018 had larger populations. A notable connection may be that 2017 was the first year in which all children 5 years of age and under had been born during the conflict, many of whom had never had access to routine services such as vaccinations. This age group was the most affected by the 2017 and 2018 epidemics (Fig. 6), and the proportion of cases for children under the age of five went up as time progressed (Fig. 5).

The four districts that faced the largest outbreaks in 2017 and 2018 accounted for 17,652 suspected cases. 3632 of these suspected cases were in 2017, while 14,020 of these suspected cases were in 2018. For comparison, if we take the average incidence of measles in 2015 and 2016 as a baseline, we would expect about 189 suspected cases across those districts affected by the 2017 outbreak and 1061 cases across those districts affected by the 2018 outbreak. This suggests that the districts affected by the 2017 outbreak experienced a 19.1-fold increase in the total number of suspected cases when compared to the previous two years, while those districts affected by the 2018 outbreak experienced a 13.2-fold increase.

Exactly what led to differences between districts in vulnerability to measles outbreaks cannot be said with certainty, but part of the explanation may be found in the geopolitical context leading up to these outbreaks. The populated regions of *Ar-Raqqa* and *Deir-Ez-Zor* fell to various opposition groups in the spring of 2013 and were primarily under *Daesh* control from January 2014 to August 2018 [49, 50]. These areas were mostly inaccessible to many aid organizations in the three years leading up to 2017 epidemic. However, the ACU was still able to operate its surveillance network in these territories, and *Daesh* even complied with the ACU’s polio campaigns in its territory [51]. It is unclear if the same was true for measles vaccination campaigns. Areas controlled by different groups may have had dramatically different access to immunizations, medications, or other services, potentially resulting in the heterogeneity of the findings.

Even in regions with robust vaccine campaigns or intact routine vaccination, vaccine hesitancy adds an additional barrier. Although formal data about vaccine distribution was not shared with us, information about these efforts was obtained during our discussions with ACU administrators. According to the members of ACU’s Rapid Response team, a branch of the ACU responsible for coordinating vaccination campaigns, vaccine hesitancy in Syria is a growing concern, especially after a tragic incident in 2014 during a measles vaccination campaign in Syria in which unlabeled vials of a muscle relaxant intended for anesthesia were accidentally administered to infants instead of the measles vaccine, leading to the deaths of 15 infants [52, 53]. This incident, coupled with rapidly spreading misinformation online, eroded trust in some communities, leading to lower vaccination rates.

What is striking is not just the overwhelming presence of cases in 2017 and 2018, but their absence in 2019. Even though we only have data for the first six months, those first six months have far less cases than any other six-month period, despite more well-established reporting mechanisms. The first six months of 2015 saw 943 suspected cases reported, and the first six months of 2016 saw 1694 suspected cases. 2017 and 2018 saw large epidemics and experienced 4398 and 15,152 suspected cases in the first six months, respectively. The first half of 2019, in contrast, had only 390 cases on record. Part of this difference may be accounted for by the changing coverage regions: as more territory fell back into the control of the Syrian government, those regions were lost to ACU surveillance coverage. Some of the most significant losses in EWARN coverage areas happened between 2018 and 2019. However, key governorates such as *Idlib*, *Ar-Raqqa*, parts of Aleppo, *Al-Hassakeh,* and *Deir-Ez-Zor* remained within EWARN’s coverage, and cases in those governorates dropped as well. Another factor is the diminished susceptible population, due to immunity acquired either by infection or vaccination. The outbreaks in 2017 and 2018, as well as strong efforts to combat them through vaccination campaigns, are likely to have contributed to shrinking the susceptible population and the relatively low measles incidence start to 2019.

### Limitations

There are several important limitations to consider for this study, given the context of conflict in which the data was collected. Among the practical limitations: we did not have access to data on weekly incidence rates for measles in Syria prior to the conflict or between the start of the conflict in March 2011 and when ACU began publishing their data in January 2015, so we cannot directly compare with pre-conflict or early conflict rates.

Furthermore, the coverage region of the ACU was contingent on the political situation on the ground. There are also some methodological limitations to the data. Although most regions have maintained high levels of completeness and timeliness of surveillance reports (estimated at greater than 95% by ACU representatives but without quantitative data available), regions that were more affected by the conflict had lower rates of completeness and timeliness, and there were regions that fell out of EWARN’s coverage.

Outbreak events initiate an investigation by the ACU, in which samples of clinically suspected cases are sent for laboratory confirmation, with a range of approximately 65–80% of suspected cases resulting in confirmation; 20–35% of suspected cases that are tested are ultimately reclassified as “discarded.” Therefore, we would expect 19,656–24,192 of the clinically suspected cases to be true laboratory confirmed cases of measles. However, these figures represent a gross underestimate of the true number of cases, since most cases of measles are unlikely to present to the clinic while others may be misdiagnosed. It is difficult to estimate how many cases there would be for every suspected case, but it would not be unreasonable to expect a manyfold increase.

While we have estimates for the positive predictive value of clinical suspicion by laboratory testing samples of clinically suspected patients, testing is still a scarce resource. Increased funding for testing would improve the quality of care for patients, as well as the precision of surveillance efforts and thus provide better data for coordinating response and research.

Furthermore, while we were able to calculate incidence based on HNAP population estimates, these values may be biased by their data collection methods, which relies on surveys, interviews, and information sharing with local organizations, or distorted due to unmeasured population movements. Future work may consider standardizing assessing population dynamics in conflict and developing standardized conflict intensity indicators^54^.

Notably, our analysis does not extend to severity of disease, morbidity, or mortality because this information is not available in our dataset. We hope this emphasizes the importance of collecting such data, which requires more support for disease surveillance in humanitarian settings.

## Conclusion

The conflict in Syria has reduced access to routine healthcare services in opposition-controlled areas, leading to 960 outbreak events, including two major epidemics of measles in 2017 and 2018 that we highlighted. The regions affected by the epidemics experienced several factors that may explain their vulnerability: these regions were heavily targeted in the conflict, they were limited in their access to aid organizations and government services, and faced vaccine hesitancy. These factors may have contributed to decreased vaccination coverage and thus increased vulnerability to widespread measles outbreaks.

While this study is not able to make causal inferences about the etiology of these outbreaks, the data suggests that these outbreaks did take place, that segments of the Syrian population have become vulnerable to vaccine-preventable diseases, and that it is possible to collect valuable, accurate, and reasonably consistent data in real-time during a conflict for response, advocacy, and research purposes. Further studies relating the severity of the conflict, intra-conflict policies and tactics, or attacks on healthcare facilities should be conducted to better understand the impact of conflict on vaccine preventable diseases. These data are crucial for rapid response coordination, advocacy, and human rights accountability. The importance of this work has only become more pronounced during SARS-CoV2 pandemic. Vaccines and other routine healthcare services are human rights. Documenting lapses in vital health services and the consequences of these lapses is an essential step in preserving those rights.

## Supplementary Information

**Additional file 1:** STROBE checklist for observational studies.

## Data Availability

Deidentified and geographically aggregated data is available on the website for Assistance Coordination Unit: https://www.acu-sy.org/en/epi-reports/. The data sets generated and/or analyzed for this study are not publicly available as their release may pose security risks for Syrian colleagues but are available from the corresponding author on reasonable request.
